# *ANGPTL4 *variants E40K and T266M are associated with lower fasting triglyceride levels in Non-Hispanic White Americans from the Look AHEAD Clinical Trial

**DOI:** 10.1186/1471-2350-12-89

**Published:** 2011-06-29

**Authors:** Melissa C Smart-Halajko, Alyson Kelley-Hedgepeth, Maria Claudia Montefusco, Jackie A Cooper, Alan Kopin, Jeanne M McCaffrey, Ashok Balasubramanyam, Henry J Pownall, David M Nathan, Inga Peter, Philippa J Talmud, Gordon S Huggins

**Affiliations:** 1 Division of Cardiovascular Genetics, British Heart Foundation Laboratories, Department of Medicine, Royal Free and UCL Medical School, London, UK; 2 MCRI Center for Translational Genomics, Molecular Cardiology Research Institute, Tufts Medical Center, Boston, MA, USA; 3 Weight Control and Diabetes Research Center, Department of Psychiatry and Human Behavior, The Miriam Hospital and Brown Medical School, Providence, RI, USA; 4 Baylor College of Medicine, Houston, TX, USA; 5 Diabetes Unit, Massachusetts General Hospital, Harvard Medical School, Boston, MA, USA; 6 Department of Genetics and Genomic Sciences, Mount Sinai School of Medicine, New York, USA

## Abstract

**Background:**

Elevated triglyceride levels are a risk factor for cardiovascular disease. Angiopoietin-like protein 4 (Angptl4) is a metabolic factor that raises plasma triglyceride levels by inhibiting lipoprotein lipase (LPL). In non-diabetic individuals, the *ANGPTL4 *coding variant E40K has been associated with lower plasma triglyceride levels while the T266M variant has been associated with more modest effects on triglyceride metabolism. The objective of this study was to determine whether ANGPTL4 E40K and T266M are associated with triglyceride levels in the setting of obesity and T2D, and whether modification of triglyceride levels by these genetic variants is altered by a lifestyle intervention designed to treat T2D.

**Methods:**

The association of *ANGPTL4 *E40K and T266M with fasting triglyceride levels was investigated in 2,601 participants from the Look AHEAD Clinical Trial, all of whom had T2D and were at least overweight. Further, we tested for an interaction between genotype and treatment effects on triglyceride levels.

**Results:**

Among non-Hispanic White Look AHEAD participants, *ANGPTL4 *K40 carriers had mean triglyceride levels of 1.61 ± 0.62 mmol/L, 0.33 mmol/L lower than E40 homozygotes (p = 0.001). Individuals homozygous for the minor M266 allele (MAF 30%) had triglyceride levels of 1.75 ± 0.58 mmol/L, 0.24 mmol/L lower than T266 homozygotes (p = 0.002). The association of the M266 with triglycerides remained significant even after removing K40 carriers from the analysis (p = 0.002). There was no interaction between the weight loss intervention and genotype on triglyceride levels.

**Conclusions:**

This is the first study to demonstrate that the *ANGPTL4 *E40K and T266M variants are associated with lower triglyceride levels in the setting of T2D. In addition, our findings demonstrate that *ANGPTL4 *genotype status does not alter triglyceride response to a lifestyle intervention in the Look AHEAD study.

## Background

Studies in large Western [1] and Asian [2] population cohorts have demonstrated an independent association between elevated triglyceride levels and cardiovascular disease (CVD) risk. Triglyceride levels may become elevated through independent effects caused by the metabolic syndrome and type 2 diabetes (T2D), both well-established risk factors for CVD [3]. Furthermore, lifestyle changes can significantly reduce triglyceride levels [4] and may moderate the risk of CVD [5].

Lipoprotein lipase (LPL) regulates triglyceride levels by hydrolyzing the triglyceride component of circulating lipoproteins [6,7]. At least some of the effects of T2D on triglycerides are mediated by LPL, whose expression and activity is influenced by insulin [6,7]. The angiopoietin-like protein 4 (Angptl4) peptide, which is primarily expressed in the liver and white adipose tissue [8], is another well established regulator of LPL activity and triglyceride levels. *In vitro *studies confirm that Angptl4, acting as an oligomer, inhibits enzymatic hydrolysis of triglycerides by preventing LPL dimerization [9]. *ANGPTL4 *has two common coding SNPs: E40K and T266M. The E40K substitution prevents Angptl4 oligomer formation, which leads to reduced Angptl4 mediated inhibition of LPL activity [10]. To date, results in over 30,000 individuals from non-diabetic [11] and population-based [12] studies have confirmed that the E40K loss-of-function variant is associated with significantly lower triglyceride levels [11,12]. In contrast, the *ANGPTL4 *T266M cSNP, which is more prevalent than E40K, has been shown to have a smaller effect on triglyceride levels in non-diabetic populations [12].

Given the importance of triglycerides and CVD risk in the context of T2D we sought to determine whether the *ANGPTL4 *E40K and T266M polymorphisms are associated with triglyceride levels in well characterized patients with T2D participating in the Look AHEAD study. Second, we asked whether the *ANGPTL4 *variants modified the triglyceride response to an intensive lifestyle intervention designed to treat T2D that was randomly assigned to Look AHEAD participants. Finding an association of ANGPTL4 variants with triglyceride levels in T2D, particularly if modifiable by a lifestyle intervention, would have important implications for personalized approaches to the treatment of T2D and cardiovascular disease.

## Methods

### Look AHEAD (Action for Health in Diabetes)

Look AHEAD is a multicenter clinical trial examining whether an intensive lifestyle intervention (ILI) aimed at weight loss and increased activity will reduce cardiovascular disease in T2D compared with diabetes support and education (DSE). The Look AHEAD multi-ethnic cohort is comprised of 5,145 male and female participants who have T2D, aged 45-76 years, and with a body mass index (BMI) ≥ 25 kg/m^2^. Included in our study are samples from Look AHEAD participants who self-identified their race and ethnicity as non-Hispanic White, African American or Hispanic. The design, baseline characteristics, and 1-year interim results have been described in detail [4,13,14]. This manuscript is based on a subset of the baseline data set from participants who provided consent for genetic studies and were enrolled from Look AHEAD sites that participated in ancillary studies (see Acknowledgement). 2,601 participants for whom DNA was available for these analyses, were genotyped for *ANGPTL4 *T266M (rs1044250, 6959 C > T) and E40K (rs116843064, 118 G > A) cSNPs using TaqMan technology (Applied Biosciences). All participants provided written informed consent for DNA collection and genetic studies as part of Look AHEAD. The Tufts Medical Center Institutional Review Board approved the study.

### Statistical Methods

Statistical analyses were performed using Intercooled Stata 10.2 for Windows (StataCorp LP, Texas, USA). A χ^2 ^test compared whether the observed frequencies conformed to Hardy-Weinberg equilibrium (HWE). A general additive model of inheritance, or dominant model in the case of E40K, was used to assess the association between genotype and clinical endpoints. All clinical data were log transformed to approximate a normal distribution before analysis. Multivariate linear regressions were used for baseline association analyses, controlling for known and significant confounders including: age, gender, study site, BMI, smoking, statins, diuretics, diabetes drugs, insulin, other lipid drugs, alcohol, menopause, and hormone replacement therapy (HRT) use. Using a dominant model there was 80% power to detect a 0.08 mmol/L difference in triglycerides between E40K allele carriers and non-carriers at baseline. Using an additive model there was 80% power to detect a difference at baseline of 0.02 mmol/L in triglyceride levels per T266M allele. Both power calculations are based on a 2-sided test at 5% significance level.

Multivariate linear regression was also utilized to determine interaction of DSE and ILI with E40K and T266M genotype. Additional association analyses performed at the 1-year follow-up time point were adjusted for age, gender, study site and the baseline measure of the dependent variable. *P *values < 0.05 were considered significant. Linkage disequilibrium (LD) between sites was estimated using Haploview (http://www.broad.mit.edu/mpg/haploview) version 3.0.

## Results

### ANGPTL4: effects on plasma Triglyceride and HDL levels in Look AHEAD

Baseline characteristics of the Look AHEAD study participants included in these analyses, separated by ethnic groups, are shown in Table 1. Of the 2,601 study participants, 1,424 are taking lipid lowering medication. *ANGPTL4 *genotype frequences are shown in Table 2. All genotype distributions were in HWE. No LD was observed between E40K and T266M in non-Hispanic White Americans (D' = 1.00 and r^2 ^= 0.05). The MAF for the E40K and T266M variants did not differ significantly between non-Hispanic Whites and Hispanics. The MAF of the E40K SNP in African Americans by comparison was very low (MAF 0.001).

**Table 1:** Baseline Data in Look AHEAD Genetic cohort by ethnic group

Variable	Non-Hispanic white Americans			African American			Hispanics			*P *value*
	n	Mean	95% CI	N	Mean	95% CI	n	Mean	95% CI	
Female (%)	2023	50.2	-	436	76.6	-	142	64.8	-	
Insulin Use (%)	2023	17.0	-	436	22.9	-	142	19.7	-	
Statin Use (%)	2023	50.1	-	436	37.6	-	142	38.0	-	
Metabolic Syndrome (%)	2023	95.4	-	436	76.6	-	142	64.8	-	
Hypertension (%)	2023	84.9	-	436	89.5	-	142	81.0	-	
Age (years)	2023	59.47	(59.17, 59.77)	436	58.40	(57.97, 58.83)	142	57.32	(56.46, 58.19)	< 0.001
HbA1c (%)	2023	7.17	(7.12, 7.21)	436	7.34	(7.27, 7.41)	142	7.52	(7.38, 7.66)	< 0.001
Weight (lbs)	2023	223.85	(222.28, 255.44)	436	220.83	(218.54, 223.14)	142	217.85	(213.36, 222.43)	0.021
BMI (kg/m2)	2023	35.88	(35.65, 36.11)	436	35.93	(35.58, 36.29)	142	35.99	(35.29, 36.70)	0.790
Waist circumference (cm)	2020	114.98	(114.42, 115.55)	436	113.17	(112.34, 114.00)	142	111.38	(109.77, 113.01)	< 0.001
Triglyceride (mmol/L)	2023	1.87	(1.82, 019)	436	1.57	(1.52, 1.62)	142	1.32	(1.23, 1.41)	< 0.001
LDL-cholesterol (mmol/L)	1975	2.77	(2.74, 2.80)	427	2.80	(2.75, 2.86)	138	2.84	(2.73, 2.94)	0.269
HDL-cholesterol (mmol/L)	1975	1.06	(1.05, 1.07)	427	1.11	(1.09, 1.13)	138	1.16	(1.13, 1.20)	< 0.001
Cholesterol (mmol/L)	1975	4.85	(4.81, 4.89)	427	4.81	(4.75, 4.87)	138	4.77	(4.66, 4.89)	0.242
Fasting Glucose (mmol/L)	1975	8.32	(8.22, 8.42)	427	8.09	(7.95, 8.24)	138	7.88	(7.60, 8.16)	0.007
SBP (mm/Hg)	1993	128.82	(128.06, 129.58)	432	129.52	(128.39, 130.66)	140	130.23	(127.98, 132.52)	0.268
DBP (mm/Hg)	1993	69.41	(69.03, 69.80)	432	70.98	(70.4, 71.57)	140	72.59	(71.41, 73.79)	< 0.001

Data presented is mean (95% confidence intervals), adjusted for age and gender;* overall comparison between the three ethnic groups after adjustment for gender.

**Table 2:** Look AHEAD genotype frequencies of ANGPTL4 E40K and T266M by ethnic group

*ANGPTL4 *variant	Genotype	Non-Hispanic White Americans			African American			Hispanic			*P *value		
		n	%	MAF	n	%	MAF	n	%	MAF	p1	p2	p3
**E40K**	**118 G > A**												
EE	GG	1868	95.8		418	99.8		129	95.6				
EK/KK	GA/AA	82/0	4.2	0.021	1/0	0.2	0.001	5/1	4.4	0.027	< 0.001	0.551	< 0.001
**T266M**	**rs1044250**												
TT	CC	961	48.1		242	56.0		63	45.0				
TM	CT	845	42.2		164	34.0		61	43.6				
MM	TT	194	9.7	0.31	26	6.0	0.25	16	11.4	0.33	< 0.001	0.403	0.007

p1; Non-Hispanic whites vs. African Americans, p2; Non-Hispanic whites vs. Hispanics, p3; African Americans vs. Hispanics.

After adjustment for covariates, the E40K variant was significantly associated with baseline plasma triglyceride levels in non-Hispanic White Look AHEAD participants (Table 3). Individuals who were K40 carriers had mean ± SD triglyceride levels of 1.61 ± 0.62 mmol/L, 0.33 mmol/L lower than E40 homozygotes (p = 0.001). A significant association of the T266M and triglyceride levels was also observed (Table 4). Individuals homozygous for the M266 allele had triglyceride levels of 1.75 ± 0.58 mmol/L, 0.24 mmol/L lower than T266 homozygotes (p = 0.002). The association of E40K and T266M with triglyceride levels remained significant in those individuals not taking a lipid lowering medication (Tables 3 and 4, respectively). The association of E40K with triglyceride levels was also significant in subjects taking lipid lowering drugs, while the triglyceride association with T266M was not found in the setting of lipid lowering therapy (data not shown).

**Table 3:** Association of E40K with baseline data in Non-Hispanic White Look AHEAD participants

	EE	EK	*P *value
	Mean (95% CI)	Mean (95% CI)	
**n males/n females**	934/934	40/42	-
**Triglycerides (mmol/L)**	1.94 (1.90, 1.99)	1.61 (1.44, 1.80)	**0.001**
**Cholesterol (mmol/L)**	4.86 (4.82, 4.90)	4.88 (4.69, 5.08)	0.889
**LDL-cholesterol (mmol/L)**	2.77 (2.73, 2.80)	2.87 (2.70, 3.04)	0.237
**HDL-cholesterol (mmol/L)**	1.05 (1.04, 1.06)	1.10 (1.05, 1.16)	0.081
**BMI (kg/m^2^)**	35.90 (35.87, 35.93)	35.91 (35.78, 36.03)	0.940
**Weight (kg)**	100.55 (100.21, 100.90)	101.51 (99.88, 103.17)	0.264
**Waist Circumference (cm)**	115.03 (114.69, 115.37)	116.73 (115.11, 118.37)	0.043
**Glucose (mmol/L)**	8.38 (8.28, 8.49)	8.02 (7.56, 8.51)	0.154
**HbA1c**	7.18 (7.14, 7.23)	7.00 (6.79, 7.22)	0.113
**SBP (mm/Hg)**	129.12 (128.31, 129.93)	124.88 (121.23, 128.64)	0.031
**DBP (mm/Hg)**	69.38 (68.98, 69.79)	67.55 (65.71, 69.43)	0.062
**Triglycerides (mmol/L) on subjects not receiving lipid lowering medication**	1.91 (1.85, 1.98) (n = 771)	1.54 (1.31, 1.80) (n = 36)	**0.007**

Data presented is mean (95% confidence intervals). Lipid data and blood pressure are adjusted for age, gender, study site, BMI, smoking, statins, diuretics, diabetes drugs, insulin, other lipid drugs, alcohol, menopause, HRT use. Anthropometric data are adjusted for age, gender, study site, smoking, statins, diuretics, diabetes drugs, insulin, other lipid drugs, alcohol, menopause, HRT use. Adjusted *P *values using a dominant model of inheritance are presented.

**Table 4:** Association of T266M with baseline data in Non-Hispanic White Look AHEAD participants

	TT	TM	MM	*P *value
	Mean (95% CI)	Mean (95% CI)	Mean (95% CI)	
**n males/n females**	461/500	428/417	93/101	-
**Triglycerides (mmol/L)**	1.99 (1.93, 2.06)	1.87 (1.81, 1.94)	1.75 (1.63, 1.89)	**0.002**
**Cholesterol (mmol/L)**	4.91 (4.85, 4.96)	4.84 (4.78, 4.90)	4.79 (4.66, 4.91)	0.120
**LDL-cholesterol (mmol/L)**	2.78 (2.73, 2.82)	2.77 (2.72, 2.83)	2.75 (2.65, 2.86)	0.937
**HDL-cholesterol (mmol/L)**	1.05 (1.04, 1.07)	1.05 (1.05, 1.12)	1.09 (1.05, 1.12)	0.178
**BMI (kg/m^2^)**	35.94 (35.90, 35.97)	35.89 (35.85, 35.93)	35.97 (35.89, 36.05)	0.101
**Weight (lbs)**	100.71 (100.22, 101.19)	100.31 (99.80, 100.83)	101.10 (100.03, 102.18)	0.331
**Waist Circumference (cm)**	115.08 (114.61, 115.55)	114.91 (114.40, 115.41)	115.77 (114.72, 116.82)	0.346
**Glucose (mmol/L)**	8.44 (8.30, 8.59)	8.32 (8.17, 8.47)	8.19 (7.89, 8.51)	0.269
**HbA1c**	7.22 (7.16, 7.29)	7.13 (7.02, 7.20)	7.08 (6.95, 7.23)	0.067
**SBP (mm/Hg)**	129.18 (128.06, 130.30)	128.68 (127.48, 129.88)	126.85 (124.41, 129.34)	0.243
**DBP (mm/Hg)**	69.45 (68.89, 70.01)	69.15 (68.55, 69.76)	68.43 (67.20, 69.68)	0.327
**Triglyceride (mmol/L)** **Following removal of E40K Carriers**	1.99 (1.92, 2.06) (n = 889)	1.88 (1.81, 1.95) (n = 775)	1.73 (1.60, 1.88) (n = 182)	**0.002**
**Triglycerides (mmol/L)** **Subjects not taking lipid-lowering medication**	1.95 (1.86, 2.05) (n = 416)	1.82 (1.74, 1.93) (n = 344)	1.66 (1.48, 1.85) (n = 75)	**0.01**

Data presented is mean (95% confidence intervals). Lipid data and blood pressure are adjusted for age, gender, study site, BMI, smoking, statins, diuretics, diabetes drugs, insulin, other lipid drugs, alcohol, menopause, HRT use. Anthropometric data are adjusted for age, gender, study site, smoking, statins, diuretics, diabetes drugs, insulin, other lipid drugs, alcohol, menopause, HRT use. Adjusted *P *values using an additive model of inheritance are presented.

We examined whether the association of T266M with triglyceride levels merely reflected the presence of E40K or whether the effect was also observed independently. When the K40 carriers were excluded, the association of T266M with triglycerides remained significant in multivariate linear regression using an additive model of inheritance, with M266 homozygotes having 0.25 mmol/L lower triglycerides compared to TT individuals (p = 0.002) (Table 4).

**Figure 1:**
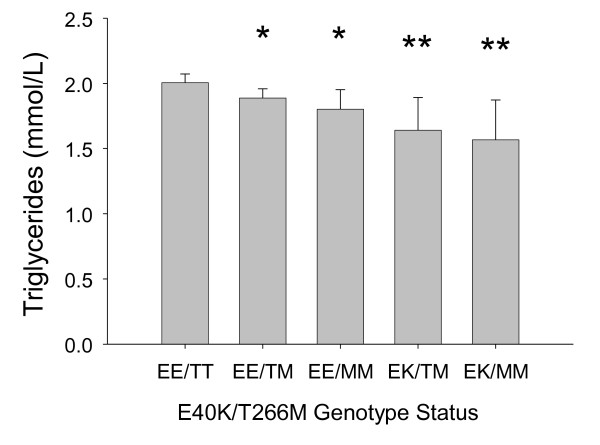
**Mean triglyceride levels in Non-Hispanic White Americans from Look AHEAD by Composite *ANGPTL4 *E40K and T266M genotype**. Triglyceride data are adjusted for age, gender, study site, BMI, smoking, statins, diuretics, diabetes drugs, insulin, other lipid drugs, alcohol, menopause, HRT use; *P *value comparing mean triglycerides to the reference EE/TT composite genotype carriers; * indicates *P *< 0.05 and ** indicates *P *< 0.01. No Look AHEAD participant was found to carry the composite EK/TT genotype.

Next, we analyzed participants by composite E40K and T266M genotypes. We found a significant genotype dosage effect in which the addition of K40 to M266 was associated with lower triglyceride levels suggesting an additive effect in the Look AHEAD cohort (Figure 1). Homozygous M266 carriers that were also heterozygous K40 carriers had the lowest triglyceride levels.

Secondary analyses of traits related to the metabolic syndrome detected a significant association of E40K with systolic blood pressure (4.24 mm/Hg lower in K40 carriers; p = 0.031) (Table 3) and a modest association with waist circumference (p = 0.043). Only triglyceride levels showed significant differences by T266M genotype (Table 4).

We were unable to test an association of E40K and triglycerides in African American and Hispanic Look AHEAD participants due to the low number of genotype carriers (Additional File 1: Supplemental Tables 1 and 2).

### One year follow-up data in non-Hispanic white Americans

Look AHEAD participants in this genetic sub-cohort randomly assigned to DSE achieved a 1% reduction of BMI compared with an 11% reduction in the ILI group (p < 0.001). The response to DSE and ILI in the Look AHEAD participants taking part in these genetic studies was similar to the one-year differences observed in the full cohort [4]. Triglyceride levels decreased in both randomisation groups, with subjects in the ILI group achieving 27% lower levels compared with 9% lower levels in the DSE group. Tests for interaction between intervention and E40K and T266M status were not statistically significant (p = 0.416 and 0.202 respectively). The absolute changes in triglyceride levels were similar between E40K and T266M genotypes (Additional File 1: Supplemental Tables 3 and 4).

## Discussion

In this study, we demonstrate for the first time that *ANGPTL4 *E40K and T266M are associated with triglyceride levels in the setting of T2D. This finding is significant because elevated triglyceride levels found in E40 homozygotes and T266 carriers may contribute to an increased risk of CVD in the setting of T2D. Non-Hispanic White Look AHEAD K40 carriers had 17% lower triglyceride levels than non-K40 carriers, which is a similar effect as reported in studies in the general population [11,12]. We therefore conclude that the effect of the *ANGPTL4 *E40K polymorphism is unlikely diminished to any substantial degree by the independent effects of obesity and T2D on triglyceride levels. The lack of interaction with treatment indicates that there is no differential effect of genotypes on triglycerides depending upon randomization to ILI versus DSE. This final point demonstrates the potential role of a life-style intervention to reduce triglyceride levels conferred by genetic background [15].

We identified an association of triglyceride levels with *ANGPTL4 *T266M that appeared to be independent of, and potentially additive with, the E40K variant. The T266M triglyceride effect is consistent with the European Atherosclerosis Research Study II CHD offspring study which demonstrated that M266 homozygotes had enhanced triglyceride clearance following an oral fat tolerance test [12]. Our results contrast to the findings of Talmud et al [12], who reported a similar pattern of linkage disequilibrium between the two cSNPs, but found the association of T266M with triglycerides to be dependent on the K40 allele. The observation that the triglyceride-lowering effect of T266M is not lost after removal of K40 carriers in Look AHEAD suggests that the T266M cSNP may have an independent effect on triglycerides in the setting of T2D. The fact that the association of T266M with triglyceride levels was not observed in Look AHEAD participants receiving lipid lowering medication, but maintained on those not on medications, suggests that an important interaction between medications and T266M may exist. Alternatively, the reduced sample size in this sub-group analysis may have reduced our power to detect an association.

E40K carriers in ARIC and the Copenhagen City Heart Study had significantly higher HDL-cholesterol levels than non-carriers, while the Dallas Heart Study did not confirm this relationship [11]. While a mechanistic link between plasma triglycerides and HDL-cholesterol mediated by the cholesterol ester transfer protein is well established [16], large differences in triglycerides are typically required before a significant change in HDL-cholesterol is found. We detected a modest, albeit non-significant, elevation of HDL-cholesterol in K40 carriers. Other factors that regulate HDL-cholesterol levels (e.g. insulin resistance, obesity) may have weakened the effect of E40K.

Both the E40 (within the N-terminus) and the T266 (within the C-terminus) amino acid residues are conserved across human, mouse and rat species [17] suggesting functional importance. Formation of Angptl4 oligomers by disulphide bonds in the coiled-coil N-terminus [17] is required for inhibition of LPL activity. The E40K substitution destabilizes the protein after secretion, preventing the extracellular accumulation of oligomers and abolishing the ability of the Angptl4 protein to inhibit LPL activity [10]. By comparison, the mechanism by which T266M alters the function of the C-terminal fibrinogen domain remains undefined. Romeo et al [18] has demonstrated that non-synonymous mutations throughout *ANGPTL4*, including the fibrinogen domain, compromise the ability of Angptl4 to inhibit LPL activity.

A limitation of the study is the low number of African American and Hispanic *ANGPTL4 *variant carriers. Statistically this resulted in underpowered association studies which prevented the examination of E40K in African Americans. The *ANGPTL4 *variant frequencies for Hispanics were comparable to Non-Hispanic White Americans (0.027 and 0.021). The low total number of Hispanics (n = 137) and E40K carriers (n = 6) reduced our power to detect an association. While we included anti-diabetic medication use as a covariate in our analyses an additional limitation is that we were unable to control for the effect of specific anti-diabetic medications and doses, which may have an effect on triglyceride levels.

## Conclusion

In conclusion, we demonstrate for the first time an association of the *ANGPTL4 *coding variants with triglyceride levels in the setting of T2D. Further, our results suggest that the triglyceride response to a lifestyle intervention is not altered by *ANGPTL4 *genotype status. This finding broadens our understanding of the role of Angptl4 in regulation of LPL metabolism of triglycerides. The independent association of T266M with triglycerides has broad implications because this polymorphism has a high frequency in the general population.

## Abbreviations

Angptl4: Angiopoietin-like protein 4; BMI: body mass index; CI: confidence intervals; CVD: cardiovascular disease; DSE: diabetes support education; HWE: Hardy-Weinberg equilibrium; HDL-cholesterol: high-density lipoprotein cholesterol; HRT-Hormone replacement therapy; ILI: intensive lifestyle intervention; LD: linkage disequilibrium; LPL: lipoprotein lipase; LDL-cholesterol: low-density lipoprotein cholesterol; MAF: minor allele frequency; SNP: single nucleotide polymorphism; cSNP: coding single nucleotide polymorphism; T2D: type 2 diabetes.

## Competing interests

The authors declare that they have no competing interests.

## Authors' contributions

MCS participated in all aspects of this project including molecular genetic studies, association analysis and manuscript preparation; AKH and IP advised association analysis and manuscript preparation; MCM and JAC carried out molecular genetic studies; AK, JM, AB, HJP, and DMN assisted in data interpretation and edited/reviewed the manuscript; PJT proposed and advised the study and edited/reviewed the manuscript; GSH participated in all aspects of the project including study design, molecular genetic studies, statistical analysis, and manuscript preparation. All authors read and approved the final manuscript.

## Pre-publication history

The pre-publication history for this paper can be accessed here:

http://www.biomedcentral.com/1471-2350/12/89/prepub

## Supplementary Material

Additional file 1 **Supplemental Analysis of ANGPTL4 E40K and T266M Genotype Status in Look AHEAD**. This additional file includes four supplemental data tables. The first two tables describe the association analysis findings of ANGPTL4 E40K (Table S1) and T266M (Table S2) with baseline biochemical and anthropometric baseline levels in African American and Hispanic Look AHEAD participants. The remaining two tables describe the association analysis findings of ANGPTL4 E40K (Table S3) and T266M (Table S4) with absolute change in baseline biochemical and anthropometric measurements to year 1 follow-up in Non-Hispanic White Look AHEAD participants. Click here for file
