# Supplementary material for: ANGPTL4 variants E40K and T266M are associated with lower fasting triglyceride levels in Non-Hispanic White Americans from the Look AHEAD Clinical Trial
Source: BMC Med Genet. 2011 Jun 29;12:89. doi: 10.1186/1471-2350-12-89 (PMC3146919; doi:10.1186/1471-2350-12-89)
Supplement: Additional file 1 — Supplemental Analysis of ANGPTL4 E40K and T266M Genotype Status in Look AHEAD. This additional file includes four supplemental data tables. The first two tables describe the association analysis findings of ANGPTL4 E40K (Table S1) and T266M (Table S2) with baseline biochemical and anthropometric baseline levels in African American and Hispanic Look AHEAD participants. The remaining two tables describe the association analysis findings of ANGPTL4 E40K (Table S3) and T266M (Table S4) with absolute change in baseline biochemical and anthropometric measurements to year 1 follow-up in Non-Hispanic White Look AHEAD participants. [file 1471-2350-12-89-S1.DOC]

**Table S1.** Association of *ANGPTL4* E40K with biochemical and anthropometric baseline data in Look AHEAD African Americans and Hispanics

|  |  | **African Americans** | | |  | **Hispanics** | | |
| --- | --- | --- | --- | --- | --- | --- | --- | --- |
|  |  | **E40K Genotype** | | |  | **E40K Genotype** | | |
|  | n | 418 | 1 | MAF 0.001 | n | 129 | 6 | MAF 0.027 |
|  |  | EE | EK/KK | p value |  | EE | EK/KK | p value |
| **Triglycerides (mmol/L)** |  | 1.303681 | 0.997451 | ND |  | 1.79557 | 1.652964 | 0.71 |
| **HDL-C (mmol/L)** |  | 1.18104 | 1.559698 | ND |  | 1.09298 | 1.029525 | 0.59 |
| **LDL-C (mmol/L)** |  | 2.896138 | 5.381502 | ND |  | 2.67288 | 2.975133 | 0.43 |
| **Cholesterol (mmol/L)** |  | 4.662 | 7.662256 | ND |  | 4.756794 | 4.927475 | 0.69 |
| **Glucose (mmol/L)** |  | 7.75668 | 7.052385 | ND |  | 8.36829 | 8.537565 | 0.88 |
| **BMI** |  | 35.79 | 43.17 | ND |  | 35.5 | 36.25 | 0.76 |

Where indicated association testing was not done (ND) for African Americans due to the low MAF. Data presented is fully adjusted for significant covariates.

**Table S2. Association of *ANGPTL4* T266M with biochemical and anthropometric baseline data in Look AHEAD African Americans and Hispanics**

|  |  | **African Americans** | | |  |  | **Hispanics** | | |  |
| --- | --- | --- | --- | --- | --- | --- | --- | --- | --- | --- |
|  |  | **T266M Genotype** | | |  |  | **T266M Genotype** | | |  |
|  | n | 242 | 164 | 26 | MAF 0.25 | n | 63 | 61 | 16 | MAF 0.33 |
|  |  | TT | TM | MM | p value |  | TT | TM | MM | p value |
| **Triglycerides (mmol/L)** |  | 1.268199 | 1.342214 | 1.349785 | 0.51 |  | 1.711046 | 1.896931 | 1.923938 | 0.49 |
| **HDL-C (mmol/L)** |  | 1.200465 | 1.171457 | 1.156435 | 0.56 |  | 1.095052 | 1.065526 | 1.171716 | 0.37 |
| **LDL-C (mmol/L)** |  | 2.923851 | 2.827762 | 3.151512 | 0.15 |  | 2.665887 | 2.688938 | 2.884483 | 0.66 |
| **Cholesterol (mmol/L)** |  | 4.67754 | 4.676245 | 4.658892 | 0.99 |  | 4.758866 | 4.803155 | 4.911417 | 0.85 |
| **Glucose (mmol/L)** |  | 7.642905 | 7.9365 | 7.813845 | 0.46 |  | 8.42157 | 8.218995 | 8.15406 | 0.86 |
| **BMI** |  | 35.88 | 35.9 | 35.49 | 0.94 |  | 35.45 | 35.72 | 36.48 | 0.83 |

Data presented is fully adjusted for significant covariates.

**Table S3. Association of *ANGPTL4* E40K with absolute change in baseline measurements to 1st Year Follow-up in Look AHEAD Non-Hispanic Whites.**

|  | **DSE** | | |  |  | **ILI** | | | |  |
| --- | --- | --- | --- | --- | --- | --- | --- | --- | --- | --- |
|  | **EE (n = 873)** | **EK/KK (n = 36)** | ***P* value** |  |  | **EE (n = 890)** | | **EK/KK (n = 41)** | ***P* value** |  |
|  | **Mean (95% CI)** | **Mean (95% CI)** |  |  |  | **Mean (95% CI)** | | **Mean (95% CI)** |  |  |
| **BMI (kg/m^2^)** | -1.18  (-1.53, -0.83) | -0.95  (-2.68, 0.78) | 0.80 |  |  | -3.61  (-3.79, -3.43) | | -3.11  (-3.94, -2.29) | 0.25 |  |
| **Glucose (mmol/L)** | -0.37  (-0.50, -0.24) | - 0.04  (-0.69, 0.62) | 0.33 |  |  | -0.49  (-0.62, -0.37) | | -0.03  (-1.29, -0.11) | 0.51 |  |
| **Cholesterol (mmol/L)** | -0.18  (-0.26, -0.11) | -0.04  (-0.44, 0.35) | 0.49 |  |  | -0.61  (-0.69, -0.54) | | -0.74  (-1.09, -0.39) | 0.48 |  |
| **Triglyceride (mmol/L)** | -0.13  (-0.18, -0.07) | -0.09  (-0.36, 0.18) | 0.78 |  |  | -0.36  (-0.42, -0.31) | | -0.51  (-0.77, -0.24) | 0.29 |  |
| **LDL-cholesterol (mmol/L)** | -0.15  (-0.19, -0.10) | -0.07  (-0.31, 0.16) | 0.54 |  |  | -0.14  (-0.18, -0.10) | | -0.16  (-0.35, 0.04) | 0.88 |  |
| **HDL-cholesterol (mmol/L)** | 0.03  (0.02, 0.04) | 0.05  (0.00, 0.11) | 0.42 |  |  | 0.09  (0.08, 0.10) | | 0.08  (0.03, 0.14) | 0.80 |  |
|  |  |  |  |  |  |  |  |  |  |  |

Data presented is mean ± 95% confidence intervals**.** Data is adjusted for age, gender, study site adjusted and adjusted for the baseline measure.

**Table S4.** Association of *ANGPTL4* T266M with absolute change in baseline measurements to 1st Year Follow-up in Look AHEAD Non-Hispanic Whites.

|  | **DSE** | | | |  | **ILI** | | | |
| --- | --- | --- | --- | --- | --- | --- | --- | --- | --- |
|  | **TT (n = 443)** | **TM (n = 397)** | **MM (n = 88)** | ***P* value** |  | **TT (n = 461)** | **TM (n = 401)** | **MM ( n = 97)** | ***P* value** |
|  | **Mean (95% CI)** | **Mean (95% CI)** | **Mean (95% CI)** |  |  | **Mean (95% CI)** | **Mean (95% CI)** | **Mean (95% CI)** |  |
| **BMI (kg/m^2^)** | -0.32  (-0.60, -0.28) | -0.40  (0.23, 0.59) | -0.52  (-0.91, -0.14) | 0.86 |  | -3.62  (-3.87, -3.37) | -3.52  (-3.79, -3.25) | -3.75  (-4.30, -3.20) | 0.73 |
| **Glucose (mmol/L)** | -0.45  (-0.63, -0.27) | -0.24  (-0.43, -0.40) | -0.41  (-0.83, 0.00) | 0.29 |  | -0.54  (-0.72, -0.36) | -0.49  (-0.68, -0.30) | -0.52  (-0.68, -0.30) | 0.95 |
| **Cholesterol (mmol/L)** | -0.22  (-0.33, -0.11) | -0.15  (-0.27, -0.04) | -0.06  (-0.31, 0.19) | 0.44 |  | -0.59  (-0.70, -0.49) | -0.64  (-0.75, -0.52) | -0.74  (-0.97, -0.51) | 0.51 |
| **Triglyceride (mmol/L)** | -0.16  (-0.23, -0.08) | -0.09  (-0.17, -0.01) | -0.17  (-0.34, 0.00) | 0.46 |  | -0.34  (-0.42, -0.27) | -0.42  (-0.51, -0.34) | -0.34  (-0.51, -0.17) | 0.39 |
| **LDL-cholesterol (mmol/L)** | -0.14  (-0.21, -0.08) | -0.11  (-0.18, -0.04) | -0.23  (-0.38, -0.08) | 0.35 |  | -0.15  (-0.21, -0.09) | -0.13  (-0.20, -0.07) | -0.19  (-0.32, -0.06) | 0.75 |
| **HDL-cholesterol (mmol/L)** | 0.03  (0.01, 0.04) | 0.04  (0.02, 0.06) | 0.04  (0.00, 0.07) | 0.37 |  | 0.10  (0.08, 0.11) | 0.09  (0.07, 0.11) | 0.07  (0.03, 0.10) | 0.33 |

Data presented is mean ± 95% confidence intervals**.** Data is adjusted for age, gender, study site adjusted and adjusted for the baseline measure.
